# Post-Induction Management in Patients With Left-Sided *RAS* and *BRAF* Wild-Type Metastatic Colorectal Cancer Treated With First-Line Anti-EGFR-Based Doublet Regimens: A Multicentre Study

**DOI:** 10.3389/fonc.2021.712053

**Published:** 2021-10-27

**Authors:** Alessandro Parisi, Alessio Cortellini, Olga Venditti, Roberto Filippi, Lisa Salvatore, Giampaolo Tortora, Michele Ghidini, Olga Nigro, Fabio Gelsomino, Ina Valeria Zurlo, Claudia Fulgenzi, Pasquale Lombardi, Susana Roselló Keränen, Ilaria Depetris, Riccardo Giampieri, Cristina Morelli, Pietro Di Marino, Francesca Romana Di Pietro, Nicoletta Zanaletti, Pasquale Vitale, Ingrid Garajova, Gian Paolo Spinelli, Federica Zoratto, Michela Roberto, Angelica Petrillo, Giacomo Aimar, Leonardo Patruno, Carla D’Orazio, Corrado Ficorella, Claudio Ferri, Giampiero Porzio

**Affiliations:** ^1^ Medical Oncology, St. Salvatore Hospital, L’Aquila, Italy; ^2^ Department of Life, Health and Environmental Sciences, University of L’Aquila, L’Aquila, Italy; ^3^ Department of Biotechnological and Applied Clinical Sciences, University of L’Aquila, L’Aquila, Italy; ^4^ Department of Surgery and Cancer, Imperial College London, London, United Kingdom; ^5^ Department of Oncology, University of Turin, Turin, Italy; ^6^ Division of Medical Oncology, Candiolo Cancer Institute, Fondazione Del Piemonte Per L'oncologia-Istiruto di Ricerca e Cura a Carattere Scientifico (FPO-IRCCS), Candiolo, Italy; ^7^ Centro Oncologico Ematologico Subalpino, Azienda Universitaria Ospedaliera Città della Salute e della Scienza di Torino, Turin, Italy; ^8^ Università Cattolica del Sacro Cuore, Rome, Italy; ^9^ Medical Oncology, Comprehensive Cancer Center, Fondazione Policlinico Universitario Agostino Gemelli, IRCCS, Rome, Italy; ^10^ Medical Oncology Unit, Fondazione IRCCS Ca’ Granda Ospedale Maggiore Policlinico, Milan, Italy; ^11^ Medical Oncology, ASST Sette Laghi, Ospedale di Circolo e Fondazione Macchi, Varese, Italy; ^12^ Department of Oncology and Hematology, Division of Oncology, University Hospital of Modena, Modena, Italy; ^13^ Medical Oncology, “Vito Fazzi” Hospital, Lecce, Italy; ^14^ Medical Oncology, Campus Bio-Medico, University of Rome, Rome, Italy; ^15^ Department of Medical Oncology, Hospital Clínico Universitario de Valencia, INCLIVA Biomedical Research Institute, University of Valencia, Valencia, Spain; ^16^ CIBERONC, Instituto de Salud Carlos III, Madrid, Spain; ^17^ Medical Oncology, ASL TO4, Ospedale Civile di Ivrea, Turin, Italy; ^18^ Clinica Oncologica e Centro Regionale di Genetica Oncologica, Università Politecnica delle Marche, AOU Ospedali Riuniti-Ancona, Ancona, Italy; ^19^ Medical Oncology Unit and PhD Program in Systems and Experimental Medicine (XXXV cycle), Tor Vergata University Hospital, Rome, Italy; ^20^ Department of Medical, Oral and Biotechnological Sciences and Center for Advance Studies and Technology (CAST), G. D’Annunzio University, Chieti, Italy; ^21^ Clinical Oncology Unit, S.S. Annunziata Hospital, Chieti, Italy; ^22^ IRCCS Istituto Dermopatico dell’Immacolata (IDI), Rome, Italy; ^23^ Experimental Clinical Abdominal Oncology Unit, Istituto Nazionale Tumori-IRCCS-Fondazione G.Pascale, Naples, Italy; ^24^ Department of Precision Medicine, Università della Campania “Luigi Vanvitelli”, Naples, Italy; ^25^ Medical Oncology Unit, University Hospital of Parma, Parma, Italy; ^26^ UOC Territorial Oncology—AUSL Latina-CdS Aprilia—University of Rome “Sapienza”, Rome, Italy; ^27^ Medical Oncology, Santa Maria Goretti Hospital, Latina, Italy; ^28^ Department of Clinical and Molecular Medicine, Sapienza University of Rome, Oncology Unit, Sant’Andrea Hospital, Rome, Italy; ^29^ Medical Oncology Unit, Ospedale del Mare, Naples, Italy

**Keywords:** MCRC, FOLFOX, FOLFIRI, cetuximab, panitumumab, maintenance, observation, de-escalation

## Abstract

**Background:**

Few data regarding post-induction management following first-line anti-epidermal growth factor receptor (EGFR)-based doublet regimens in patients with left-sided *RAS/BRAF* wild-type metastatic colorectal cancer (mCRC) are available.

**Methods:**

This multicenter, retrospective study aimed at evaluating clinicians’ attitude, and the safety and effectiveness of post-induction strategies in consecutive patients affected by left-sided *RAS/BRAF* wild-type mCRC treated with doublet chemotherapy plus anti-EGFR as first-line regimen, who did not experience disease progression within 6 months from induction initiation, at 21 Italian and 1 Spanish Institutions. The measured clinical outcomes were: progression-free survival (PFS), overall survival (OS), adverse events, and objective response rate (ORR).

**Results:**

At the data cutoff, among 686 consecutive patients with left-sided *RAS/BRAF* wild-type mCRC treated with doublet plus anti-EGFR as first-line regimen from March 2012 to October 2020, 355 eligible patients have been included in the present analysis. Among these, 118 (33.2%), 66 (18.6%), and 11 (3.1%) received a maintenance with 5-fluorouracil/leucovorin (5FU/LV)+anti-EGFR, anti-EGFR, and 5FU/LV, respectively, while 160 (45.1%) patients continued induction treatment (non-maintenance) until disease progression, unacceptable toxicity, patient decision, or completion of planned treatment. The median period of follow-up for the overall population was 33.7 months (95%CI = 28.9–35.6). The median PFS values of the 5FU/LV+anti-EGFR, anti-EGFR, 5FU/LV, and non-maintenance cohorts were 16.0 (95%CI = 14.3–17.7, 86 events), 13.0 (95%CI = 11.4–14.5, 56 events), 14.0 (95%CI = 8.1–20.0, 8 events), and 10.1 months (95%CI = 9.0–11.2, 136 events), respectively (*p* < 0.001). The median OS values were 39.6 (95%CI = 31.5–47.7, 43 events), 36.1 (95%CI = 31.6–40.7, 36 events), 39.5 (95%CI = 28.2–50.8, 4 events), and 25.1 months (95%CI = 22.6–27.6, 99 events), respectively (*p* < 0.001). After adjusting for key covariates, a statistically significant improvement in PFS in favor of 5FU/LV+anti-EGFR (HR = 0.59, 95%CI = 0.44–0.77, *p* < 0.001) and anti-EGFR (HR = 0.71, 95%CI = 0.51–0.98, *p* = 0.039) compared to the non-maintenance cohort was found. Compared to the non-maintenance cohort, OS was improved by 5FU/LV+anti-EGFR (HR = 0.55, 95%CI = 0.38–0.81, *p* = 0.002) and, with marginal significance, by anti-EGFR (HR = 0.67, 95%CI = 0.51–0.98, *p* = 0.051). No difference was found in ORR. Any grade non-hematological and hematological events were generally higher in the non-maintenance compared to the maintenance cohorts.

**Conclusion:**

Among the treatment strategies following an anti-EGFR-based doublet first-line induction regimen in patients affected by left-sided *RAS/BRAF* wild-type mCRC treated in a “real-life” setting, 5FU/LV+anti-EGFR resulted the most adopted, effective, and relatively safe regimen.

## Introduction

The introduction of biological agents and the development of continuum of care strategies profoundly changed the treatment landscape for patients with unresectable metastatic colorectal cancer (mCRC). As the maximum benefit is achieved during the first-line treatment, strategies to consolidate the obtained response, maintaining the disease control while keeping a good safety profile, are essential. This applies even more with oxaliplatin-based regimens, as peripheral neuropathy could strongly worsen the long-term quality of life of patients (1). The landmark randomized, phase 3 OPTIMOX1 study found no difference in progression-free survival (PFS), overall survival (OS), and objective response rate (ORR) between a maintenance strategy with fluorouracil/leucovorin (5FU/LV) and full chemotherapy continuation after six induction cycles of 5FU/LV and oxaliplatin (FOLFOX). The better safety profile of the de-escalated arm, including a lower incidence of grade 3–4 cumulative peripheral sensory neuropathy, led to a progressive change in clinical practice by adopting maintenance strategies with 5FU/LV in association with a targeted agent (1).

Multiple phase 3 studies have investigated the role of maintenance anti-vascular endothelial growth factor (VEGF) blockade with bevacizumab/fluoropyrimidine following induction chemotherapy in the first-line setting, with variable benefits in terms of PFS and a good safety profile compared to no de-escalation and treatment holidays (2–5). According to these results and current guidelines, bevacizumab plus a fluoropyrimidine is regarded as the optimal maintenance regimen after a 4- to 6-month induction treatment with bevacizumab plus doublet or triplet regimens (6).

An anti-epidermal growth factor receptor (EGFR) agent (i.e., cetuximab or panitumumab) added to doublet chemotherapy is currently recommended as the first-line treatment option, particularly in left-sided *RAS/BRAF* wild-type mCRC (6–8). However, only few phase 2 studies investigating the role of maintenance (9–12) or intermittent (13, 14) strategies following anti-EGFR-based induction are available. The aim of this study was to retrospectively assess clinicians’ attitude and the safety and effectiveness of anti-EGFR post-induction strategies in a “real-life” population of patients affected by unresectable left-sided *RAS/BRAF* wild-type mCRC.

## Materials and Methods

### Patient Selection

This retrospective analysis evaluated consecutive unresectable *RAS* and *BRAF* wild-type left-sided mCRC patients treated with first-line doublet chemotherapy plus an anti-EGFR agent outside of a clinical trial setting at 21 Italian and 1 Spanish institutions (**Supplementary File 1**) from March 2012 to October 2020.

**Figure 1:**
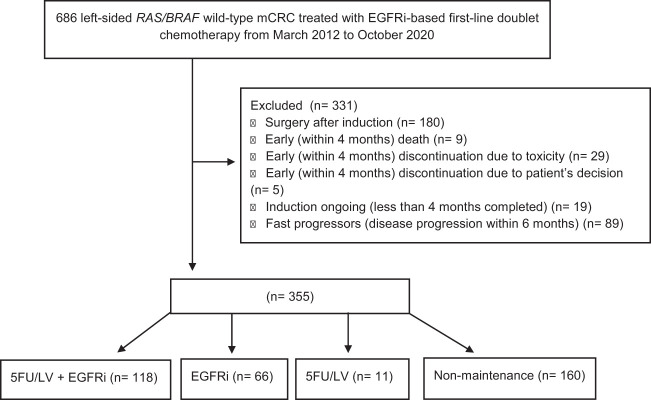
CONSORT flowchart of patient selection and disposition.

The eligibility criteria were: age ≥18 years; histologically confirmed diagnosis of CRC originating from the splenic flexure, descending colon, sigma, and rectum; confirmed *KRAS* (exons 2–4), *NRAS* (exons 2–4), and *BRAF* (V600E) wild-type genotype; and having received a first-line treatment with an anti-EGFR-based doublet [FOLFOX or irinotecan/5-fluorouracil/leucovorin (FOLFIRI)]. The exclusion criteria were: surgery after an induction treatment; early (within 4 months) discontinuation of the induction due to death, toxicity, or patient’s decision; induction treatment ongoing (defined as less than 4 months treatment completed) at the time of data cutoff analysis; or fast progressors (i.e., patients who experienced disease progression within 6 months from the beginning of the induction treatment). The CONSORT flow diagram with patient selection is presented in **Figure 1**.

All patients alive at the time of data collection provided informed consent to participate in this retrospective, observational, non-interventional study. The procedures followed were in accordance with the precepts of Good Clinical Practice and the Declaration of Helsinki. The study was approved by the respective local ethical committees on human experimentation of each institution, after previous approval by the coordinating center (Comitato Etico delle Province di L’Aquila e Teramo, protocol no, 21, approved on July 16, 2020).

### Study Design

The measured effectiveness, safety, and antitumor activity clinical outcomes were PFS, OS, ORR, and treatment-related adverse events (AEs). Disease responses were evaluated with the RECIST (Response Evaluation Criteria in Solid Tumors) (version 1.1) (15). Only patients with measurable disease at the time of first radiological assessment were included in the activity analysis. ORR was defined as the portion of patients experiencing an objective response (complete response or partial response) as best response. PFS was defined as the length of time from the beginning of first-line treatment to disease progression or death from any cause; OS, as the length of time between the beginning of first-line treatment to death from any cause. Data cutoff period was October 2020. For the study purpose, we grouped patients according to the type of maintenance treatment, if received, regardless of the duration of the induction period: 5FU/LV+anti-EGFR, anti-EGFR, 5FU/LV, and non-maintenance (i.e., induction continuation). The baseline characteristics of patients were compared across the four cohorts.

Fixed regression models were used for the multivariable analyses of PFS and OS. Covariates were chosen with a clinical prioritization approach and on the basis of their availability (16–18). The chosen key covariates were: age (<70 *vs*. ≥70 years) (19), gender (male *vs*. female) (20), Eastern Cooperative Oncology Group—Performance Status (ECOG-PS) (0 *vs*. 1–2), number of metastatic sites (one *vs*. two or more), baseline alkaline phosphatase (ALP; normal *vs*. high), and white blood cell (WBC) count (normal *vs*. high) (21).

AEs experienced during induction and maintenance treatments were clustered as: hematological (leukopenia, anemia, and thrombocytopenia); non-hematological (nausea, vomiting, mucositis, hand–foot syndrome, asthenia, anorexia, and others); and anti-EGFR class-specific AEs (skin rash/acneiform dermatitis, paronychia/nail disorders, and others). Because of their clinical relevance, diarrhea, peripheral neuropathy, and neutropenia were evaluated individually. AEs were reported for the overall population, registered according to National Cancer Institute Common Terminology Criteria (NCI-CTC) for AEs (version 4 up to January 2018 and version 5 from January 2018), and grouped according to severity (G1–2 and G3–4). In the non-maintenance cohort, AEs have been collected throughout the entire duration of treatment.

### Statistical Analysis

Chi-square and Fisher’s exact tests, as appropriate, were used to compare the baseline characteristics of patients, reported with descriptive statistics, and treatment outcomes across the cohorts. Survival analysis employed the Kaplan–Meier method, in which patients without events were censored at the last follow-up available, and log-rank test for inter-cohort comparisons. The Cox proportional hazard model was used for the univariate and multivariate analyses and for calculating hazard ratios (HRs) with 95% confidence intervals (CIs). The median period of follow-up was calculated through the reverse Kaplan–Meier method. The threshold for statistical significance was set to *p* = 0.05. All statistical analyses were performed using IBM SPSS Statistics, version 26.0 (released 2019, IBM SPSS Statistics for Macintosh, version 26.0; IBM Corp., Armonk, NY, USA).

### Molecular Profile Assessment

All the molecular analyses were performed according to the local clinical practice of the participating centers. *KRAS*, *NRAS*, and *BRAF* mutational status was assessed with Sanger sequencing, real-time PCR techniques, and next-generation sequencing (NGS) (such as OncoGenBasic-S1 kit, Seqplexing; Pyromark Q96 ID System, Qiagen; EasyPGX and Myriapod Colon Status, Diatech Pharmacogenetics; Idylla KRAS and NRAS-BRAF Mutation Test, Biocartis; and Ion AmpliSeq Colon and Lung Cancer Panel, Ion Torrent).

## Results

### Patient Characteristics

At the data cutoff, the clinical histories of 686 consecutive patients with left-sided *RAS* and *BRAF* wild-type mCRC treated with doublet plus anti-EGFR as first-line regimen were entered. After the exclusion of 331 patients, 355 eligible patients have been included in the present analysis (**Figure 1**). Among these, 118 (33.2%), 66 (18.6%), and 11 (3.1%) received a maintenance regimen with 5FU/LV+anti-EGFR, anti-EGFR, and 5FU/LV, respectively; meanwhile, 160 (45.1%) patients continued induction treatment (non-maintenance) until completion of 4–6 months of planned treatment (i.e., “stop-and-go” or intermittent approach), disease progression, unacceptable toxicity, or patient decision. Patients’ features are summarized in **Table 1**. The median age was 64 years (range = 29–84). A statistically significant difference was found between the four cohorts with respect to disease burden, as a higher number of metastatic sites was found in the 5FU/LV+anti-EGFR (52.5%), anti-EGFR (55.4%), and non-maintenance (60.3%) cohorts compared to that in the 5FU/LV (18.2%) cohort. Moreover, statistically significant differences were found with regard to the chemotherapy induction backbone and the anti-EGFR used. Within the non-maintenance cohort, 90 (56.2%) patients were treated up to disease progression, unacceptable toxicity, or drug holiday/patient decision. Among these, 31 (34.4%) and 51 (65.6%) patients were treated with FOLFOX or FOLFIRI, respectively, in association with panitumumab (39, 43.3%) or cetuximab (51, 56.7%). Within the non-maintenance cohort, 70 (43.7%) patients were treated with a “stop-and-go” strategy with FOLFOX (39, 55.7%) or FOLFIRI (31, 44.3%) in association with panitumumab (41, 58.6%) or cetuximab (29, 41.4%).

**Table 1:** Baseline demographic and disease characteristics.

	Overall (*n* = 355), *N* (%)	5-FU/LV+anti-EGFR (*n* = 118), *N* (%)	Anti-EGFR (*n* = 66), *N* (%)	5-FU/LV (*n* = 11), *N* (%)	Non-maintenance (*n* = 160), *N* (%)	*χ* ^2^ test (*p*-value)
Age (years)						
Median	64	64	66	68	63	0.670
Range	29–84	29–81	39–81	50–76	31–84	
<70 years	255 (71.8)	82 (69.5)	45 (68.2)	8 (72.7)	120 (75.0)	
≥70 years	100 (28.2)	36 (30.5)	21 (31.8)	3 (27.3)	40 (25.0)	
Gender						
Male	215 (60.6)	74 (62.7)	42 (63.6)	3 (27.3)	96 (60.0)	0.132
Female	140 (39.4)	44 (37.3)	24 (36.4)	8 (72.7)	64 (40.0)	
ECOG-PS						
0	220 (62.0)	75 (63.6)	39 (59.1)	7 (63.6)	99 (61.9)	0.946
1–2	135 (38.0)	43 (36.4)	27 (40.9)	4 (36.4)	61 (38.1)	
Previous adjuvant treatment						
None	266 (74.9)	93 (78.8)	53 (80.3)	8 (72.7)	112 (70.0)	0.603
Fluoropyrimidine alone	51 (14.4)	15 (12.7)	8 (12.1)	2 (18.2)	26 (16.3)	
XELOX/FOLFOX	38 (10.7)	10 (8.5)	5 (7.6)	1 (9.1)	22 (13.8)	
No. of metastatic sites						
1	160 (45.1)	56 (47.5)	29 (44.6)	9 (81.8)	62 (39.7)	0.017
≥2	195 (54.9)	62 (52.5)	36 (55.4)	2 (18.2)	94 (60.3)	
ALP^a^						
Normal	271 (78.6)	94 (80.3)	51 (82.3)	11 (100.0)	115 (74.2)	0.140
High	79 (21.4)	23 (19.7)	11 (17.7)	0 (0.0)	40 (25.8)	
WBC^b^						
Normal	229 (66.2)	84 (71.8)	42 (66.7)	10 (90.9)	93 (60.0)	0.063
High	117 (33.8)	33 (28.2)	21 (33.3)	1 (9.1)	62 (40.0)	
Time to metastases						
Metachronous	94 (26.5)	26 (22.0)	19 (28.8)	3 (27.3)	46 (28.7)	0.614
Synchronous	261 (73.5)	92 (78.0)	47 (71.2)	8 (72.7)	114 (71.3)	
Chemotherapy backbone						
FOLFOX	188 (53.0)	77 (65.3)	32 (48.5)	9 (81.8)	70 (43.8)	0.001
FOLFIRI	167 (47.0)	41 (34.7)	34 (51.5)	2 (18.2)	90 (56.3)	
Anti-EGFR						
Panitumumab	194 (54.6)	80 (67.8)	26 (39.4)	8 (72.7)	80 (50.0)	0.001
Cetuximab	161 (45.4)	38 (32.2)	40 (60.6)	3 (27.3)	80 (50.0)	

5-FU/LV, 5-fluorouracil/leucovorin; EGFR, epidermal growth factor receptor; ECOG, Eastern Cooperative Oncology Group—Performance Status; XELOX, capecitabine plus oxaliplatin; FOLFOX, 5FU/LV and oxaliplatin; FOLFIRI, irinotecan/5-fluorouracil/leucovorin; ALP, alkaline phosphatase; WBC, white blood cell.

a Thirteen patients not evaluable.

b Four patients not evaluable.

### Clinical Outcome Analysis

The median period of follow-up for the overall population was 33.7 months (95%CI = 28.9–35.6), while those among the 5FU/LV+anti-EGFR, anti-EGFR, 5FU/LV, and non-maintenance cohorts were 26.4 (95%CI = 18.1–34.7), 42.0 (95%CI = 33.6–50.4), 30.0 (95%CI = 13.7–46.3), and 38.3 months (95%CI = 27.6–49.0), respectively.

**Figure 2:**
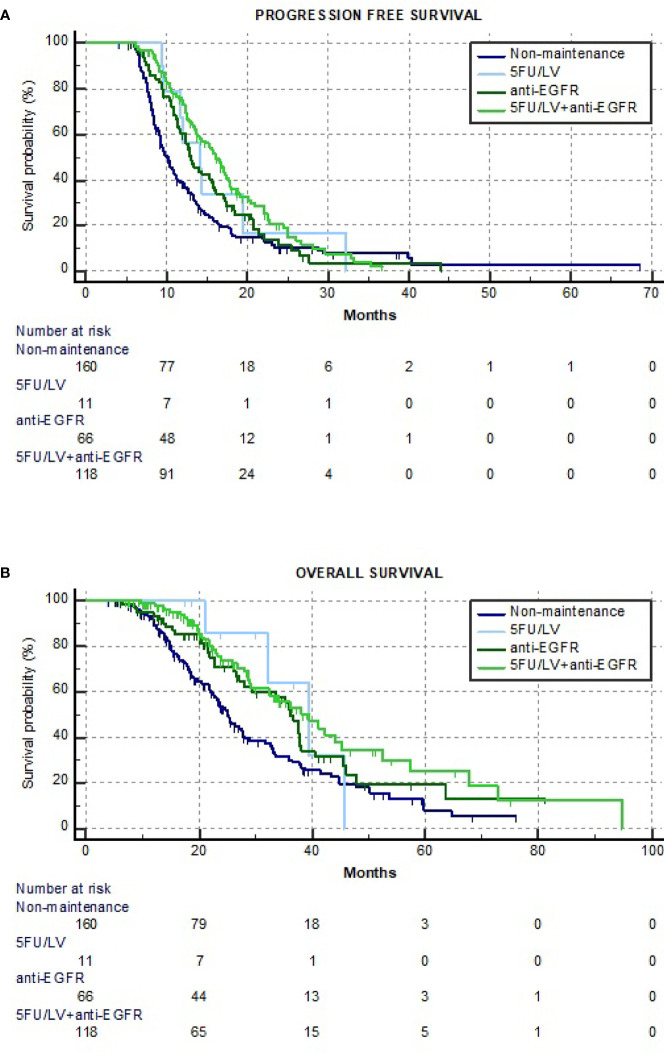
Kaplan–Meier estimate curves of progression-free survival (PFS) **(A)** and overall survival (OS) **(B)**.

The median PFS of the overall population was 12.6 months (95%CI = 11.8–13.4, 286 events), while the median PFS values of the 5FU/LV+anti-EGFR, anti-EGFR, 5FU/LV, and non-maintenance cohorts were 16.0 (95%CI = 14.3–17.7, 86 events), 13.0 (95%CI = 11.4–14.5, 56 events), 14.0 (95%CI = 8.1–20.0, 8 events), and 10.1 months (95%CI = 9.0–11.2, 136 events), respectively, with a statistically significant heterogeneity at the univariate analysis (*p* < 0.001). The median OS of the overall population was 32.3 months (95%CI = 27.7–36.7, 182 events), while median OS values of the 5FU/LV+anti-EGFR, anti-EGFR, 5FU/LV, and non-maintenance cohorts were 39.6 (95%CI = 31.5–47.7, 43 events), 36.1 (95%CI = 31.6–40.7, 36 events), 39.5 (95%CI = 28.2–50.8, 4 events), and 25.1 months (95%CI = 22.6–27.6, 99 events), respectively, with a statistically significant heterogeneity at the univariate analysis (*p* < 0.001) (**Table 2** and **Figure 2**). After adjusting for the key covariates, a statistically significant improvement in PFS was found in the multivariate analysis in favor of 5FU/LV+anti-EGFR (HR = 0.59, 95%CI = 0.44–0.77, *p* < 0.001) and anti-EGFR (HR = 0.71, 95%CI = 0.51–0.98, *p* = 0.039) compared to the non-maintenance cohort. Moreover, a statistically significant improvement in OS was found at the multivariate analysis in favor of 5FU/LV+anti-EGFR (HR = 0.55, 95%CI = 0.38–0.81, *p* = 0.002), while a trend toward better OS was found for anti-EGFR (HR = 0.67, 95%CI = 0.51–0.98, *p* = 0.051) compared to the non-maintenance cohort (**Table 3**).

**Table 2:** Treatment outcomes during first-line treatment.

	5-FU/LV+anti-EGFR (*n* = 118)	Anti-EGFR (*n* = 66)	5-FU/LV (*n* = 11)	Non-maintenance (*n* = 160)	*p*-value
Median OS, *n* (95%CI) [events]	39.6 (31.5–47.7) [43]	36.1 (31.6–40.7) [36]	39.5 (28.2–50.8) [4]	25.1 (22.6–27.6) [99]	<0.001 (log-rank)
Median PFS, *n* (95%CI) [events]	16.0 (14.3–17.7) [86]	13.0 (11.4–14.5) [56]	14.0 (8.1–20.0) [8]	10.1 (9.0–11.2) [136]	<0.001(log-rank)
Median no. of induction cycles (range)	12 (6–15)	12 (6–18)	11 (8–13)	12 (6–36)	–
Median no. of maintenance cycles (range)	11 (1–51)	11 (2–78)	7 (2–9)	–	–
Response/ratio (ORR, %) during induction^a^	92/118 (78.0)	50/63 (79.4)	9/11 (81.8)	112/157 (71.3)	0.459 (*χ* ^2^ test)
10-month PFS (%)	77.1	72.7	63.6	48.1	<0.001 (*χ* ^2^ test)
Cause of induction discontinuation^b^, *N* (%)					
Toxicity Disease progression Planned treatment completed Patient decision/drug holiday	11 (9.3) 6 (5.1) 95 (80.5) 6 (5.1)	5 (7.6) 1 (1.5) 60 (90.9) 0 (0.0)	1 (9.1) 0 (0.0) 9 (81.8) 1 (9.1)	13 (8.1) 69 (43.1) 70 (43.8) 8 (5.0)	<0.001 (*χ* ^2^ test)
Cause of maintenance discontinuation, *N* (%)					
Toxicity Disease progression Patient decision/drug holiday Loss to follow-up Treatment ongoing Surgery or locoregional treatment Complete response/NED Planned treatment completed	6 (5.5) 75 (68.2) 6 (5.5) 0 (0.0) 21 (19.1) 1 (0.9) 0 (0.0) 1 (0.9)	4 (6.9) 45 (77.6) 3 (5.2) 2 (3.4) 3 (5.2) 0 (0.0) 1 (1.7) 0 (0.0)	1 (9.1) 7 (63.6) 0 (0.0) 0 (0.0) 1 (9.1) 1 (9.1) 1 (9.1) 0 (0.0)	–	0.023 (*χ* ^2^ test)
Second line treatment, *N* (%)					
Mono/doublet+bevacizumab FOLFIRI+aflibercept Mono/doublet+anti-EGFR reintro Other	44 (57.9) 20 (26.3) 6 (7.9) 6 (7.9)	35 (71.4) 7 (14.3) 3 (6.1) 4 (8.2)	2 (33.3) 1 (16.7) 1 (16.7) 2 (33.3)	71 (60.7) 10 (8.5) 18 (15.4) 18 (15.4)	<0.023 (*χ* ^2^ test)

Reintro, reintroduction; 5-FU/LV, 5-fluorouracil/leucovorin; EGFR, epidermal growth factor receptor; OS, overall survival; PFS, progression-free survival; ORR, overall response rate; NED, no evidence of disease; FOLFIRI, irinotecan/5-fluorouracil/leucovorin.

a Computed among evaluable patients only.

b In the non-maintenance cohort, the definition of “induction” included all patients who continued treatment (for 6 months or more) until discontinuation for any reason (i.e., toxicity, progressive disease, completion of planned treatment, or patient decision/drug holiday).

**Table 3:** Univariate and multivariate analyses for progression-free survival and overall survival.

Variable (comparator)	Progression-free survival				Overall survival			
	Univariate		Multivariate		Univariate		Multivariate	
	HR (95%CI)	*p*-value	HR (95%CI)	*p*-value	HR (95%CI)	*p*-value	HR (95%CI)	*p*-value
Treatment (none)								
5FU+anti-EGFR	0.55 (0.42–0.73)	<0.001	0.59 (0.44–0.77)	<0.001	0.49 (0.34–0.70)	<0.001	0.55 (0.38–0.81)	0.002
Anti-EGFR	0.73 (0.53–0.99)	0.048	0.71 (0.51–0.98)	0.039	0.64 (0.43–0.93)	0.021	0.67 (0.45–1.01)	0.051
5FU	0.63 (0.31–1.28)	0.629	0.75 (0.36–1.56)	0.435	0.50 (1.83–1.35)	0.171	0.78 (0.28–2.20)	0.641
WBC count (normal)								
High	1.42 (1.11–1.82)	0.006	1.31 (0.99–1.73)	0.062	1.87 (1.38–2.53)	<0.001	1.55 (1.11–2.19)	0.011
ALP (normal)								
High	1.30 (0.97–1.75)	0.076	1.07 (0.77–1.49)	0.691	1.87 (1.33–2.63)	<0.001	1.35 (0.92–1.97)	0.122
ECOG-PS (0)								
1–2	1.13 (0.89–1.43)	0.387	1.07 (0.83–1.37)	0.619	1.43 (1.07–1.92)	0.016	1.17 (0.85–1.61)	0.342
No. of met. sites (1)								
≥2	1.31 (1.03–1.65)	0.025	1.20 (0.93–1.53)	0.158	1.63 (1.20–2.21)	0.002	1.39 (1.01–1.92)	0.046
Sex (male)								
Female	1.01 (0.80–1.28)	0.924	1.02 (0.80–1.3)	0.891	0.88 (0.65–1.18)	0.389	0.83 (0.61–1.14)	0.254
Age (non-elderly)								
Elderly (≥70 years)	0.99 (0.77–1.29)	0.984	1.10 (0.83–1.45)	0.515	1.32 (0.95–1.83)	0.093	1.43 (1.01–2.02)	0.047

5-FU, 5-fluorouracil; EGFR, epidermal growth factor receptor; ECOG, Eastern Cooperative Oncology Group—Performance Status; ALP, alkaline phosphatase; WBC, white blood cell.

The ORRs were 78% (95%CI = 69.9–84.7), 79.4% (95%CI = 68.2-87.9), 81.8% (95%CI = 53.3–96), and 71.3% (95%CI = 63.9–78.0) in the 5FU/LV+anti-EGFR, anti-EGFR, 5FU/LV, and non-maintenance (*p* = 0.459) cohorts, respectively (**Table 2**).

### Safety Analysis

The toxicity profiles are summarized in **Table 4**. The AEs that occurred most commonly during maintenance treatment with 5FU/LV+anti-EGFR, anti-EGFR, and 5FU/LV were any grade non-hematological (24.6%, 9.1%, and 27.3%, respectively), hematological (22.9%, 7.6%, and 27.3%, respectively), neutropenia (20.3%, 7.6%, and 9.1%, respectively), skin rash (65.3%, 68.2%, and 9.1%, respectively), and paronychia/nail disorders (33.1%, 19.7%, and 0.0%, respectively). Among the G3–G4 AEs, diarrhea was more frequent in the 5FU/LV cohort (9.1%), while skin rash was more frequent in the 5FU/LV+anti-EGFR and anti-EGFR cohorts (8.5% and 9.1%, respectively). In general, the non-maintenance cohort had higher incidence rates of any grade non-hematological and hematological AEs, diarrhea, and neutropenia compared to those of the 5FU/LV+anti-EGFR and anti-EGFR cohorts.

**Table 4:** Induction and maintenance of treatment-related adverse events (AEs).

	5-FU/LV+anti-EGFR (*n* = 118)			Anti-EGFR (*n* = 66)		5-FU/LV (*n* = 11)		Non-maintenance (*n* = 160)^a^	
	Any grade	G3–G4		Any grade	G3–G4	Any grade	G3–G4	Any grade	G3–G4
Induction AEs, *n* (%)									
Non-hematological^b^	43 (36.4)	2 (1.7)		15 (22.7)	0 (0.0)	6 (54.5)	0 (0.0)	82 (51.2)	11 (6.9)
Diarrhea	44 (37.3)	2 (1.7)		20 (30.3)	2 (3.0)	4 (36.4)	1 (9.1)	88 (55.0)	8 (5.0)
Peripheral neuropathy^c^	60 (50.8)	7 (5.9)		16 (24.2)	2 (3.0)	7 (63.6)	0 (0.0)	59 (36.9)	6 (3.8)
Hematological^d^	43 (36.4)	2 (1.7)		15 (22.7)	0 (0.0)	6 (54.5)	0 (0.0)	82 (51.2)	11 (6.9)
Neutropenia	52 (44.1)	16 (13.6)		20 (30.3)	6 (9.1)	6 (54.5)	3 (27.3)	83 (51.9)	22 (13.8)
Skin rash	103 (87.3)	26 (22.0)		46 (69.7)	7 (10.6)	8 (72.7)	1 (9.1)	134 (83.8)	35 (21.9)
Paronychia/nail disorders^e^	60 (50.8)	5 (4.2)		16 (24.2)	2 (3.0)	6 (54.5)	1 (9.1)	74 (46.3)	8 (5.0)
Other anti-EGFR-related^f^	21 (17.8)	2 (1.7)		10 (15.2)	2 (3.0)	1 (9.1)	0 (0.0)	41 (25.6)	6 (3.8)
Maintenance AEs, *n* (%)									
Non-hematological^b^	29 (24.6)		0 (0.0)	6 (9.1)	0 (0.0)	3 (27.3)	0 (0.0)	–	
Diarrhea	20 (16.9)		2 (1.7)	9 (13.6)	0 (0.0)	1 (9.1)	1 (9.1)	–	
Hematological^d^	27 (22.9)		2 (1.7)	5 (7.6)	0 (0.0)	3 (27.3)	0 (0.0)	–	
Neutropenia	24 (20.3)		0 (0.0)	5 (7.6)	1 (1.5)	1 (9.1)	0 (0.0)	–	
Skin rash	77 (65.3)		10 (8.5)	45 (68.2)	6 (9.1)	1 (9.1)	0 (0.0)	–	
Paronychia/nail disorders	39 (33.1)		2 (1.7)	13 (19.7)	0 (0.0)	0 (0.0)	0 (0.0)	–	
Other anti-EGFR-related^e^	14 (11.9)		1 (0.8)	10 (15.2)	1 (1.5)	0 (0.0)	0 (0.0)	–	

5-FU/LV, 5-fluorouracil/leucovorin; EGFR, epidermal growth factor receptor; AEs, adverse events.

a In the non-maintenance cohort, AEs have been collected throughout the entire duration of treatment.

b Diarrhea and peripheral neuropathy excluded.

c Among patients treated with oxaliplatin.

d Neutropenia excluded.

e Periungueal pyogenic granuloma, fissures, onycholysis, and others.

f Hypomagnesemia, dry skin, pruritus, conjunctivitis, mucositis, and others.

### Induction and Maintenance Discontinuation and Post-Progression Treatments

Completion of the planned induction treatment was achieved by 80.5%, 90.9%, and 81.8% of patients in the 5FU/LV+anti-EGFR, anti-EGFR, and 5FU/LV cohorts, respectively. Among them, 68.2%, 77.6%, and 63.6%, respectively, discontinued the maintenance treatment due to disease progression. On the other hand, 43.1% and 43.8% of patients in the non-maintenance cohort discontinued the induction treatment due to disease progression and completion of planned treatment, respectively.

As expected, most of the patients underwent an antiangiogenic-containing second-line regimen with bevacizumab or aflibercept, while a lower rate of patients was treated with the anti-EGFR reintroduction in association with a mono- or doublet chemotherapy (**Table 2**).

## Discussion

Compared to bevacizumab-based strategies, anti-EGFR-based post-induction treatment options are less codified and no phase 3 data are available.

According to the results of phase 2 trials, anti-EGFR-based maintenance therapy is feasible in mCRC patients after oxaliplatin-based induction regimens.

The randomized, phase 2 MACRO-2 study compared continued treatment with FOLFOX–cetuximab *vs*. maintenance cetuximab after induction with eight cycles of FOLFOX–cetuximab in *KRAS* wild-type mCRC Western patients. No difference was found between the continued oxaliplatin and maintenance groups in terms of PFS (9.8 *vs*. 8.7 months, respectively), with reduced incidence of peripheral neuropathy (15% *vs*. 2%, respectively) and acneiform rash (24% *vs*. 15%, respectively) (11).

The randomized, phase 2 SAPPHIRE study compared continued treatment with FOLFOX+panitumumab *vs*. 5FU/LV+panitumumab after induction with six cycles of FOLFOX+panitumumab in *RAS* wild-type mCRC Eastern patients. The median PFS was comparable between the continued oxaliplatin group and the de-escalated group (9.1 and 9.3 months, respectively), with slightly improved outcomes in left-sided patients (10.5 *vs*. 11.5 months, respectively) and a reduced incidence of peripheral neuropathy (13.5% *vs*. 1.9%, respectively) (12).

In the non-comparative phase 2 COIN-B study, *KRAS* exon 2 wild-type mCRC patients were randomized to receive FOLFOX–cetuximab for 12 weeks followed by cetuximab maintenance *vs*. observation and reintroduced FOLFOX–cetuximab at disease progression. No difference was noted among the maintenance and intermittent strategies in 10-month failure-free survival (52% *vs*. 50%, respectively), even if a trend toward better post-induction PFS (5.8 *vs*. 3.1 months, respectively) and OS (22.2 *vs*. 16.8 months, respectively) was observed in favor of the maintenance treatment, particularly in the *RAS* wild-type population (13).

In the randomized phase 2 VALENTINO study, *RAS* wild-type mCRC patients were randomized to receive induction with FOLFOX–panitumumab for eight cycles, followed by either 5FU/LV+panitumumab or panitumumab alone as maintenance. A clinically relevant benefit in favor of 5FU/LV+panitumumab in terms of 10-month PFS (59% *vs*. 49%) and median PFS (12 *vs*. 9.9 months) was observed. As expected, a higher incidence of AEs, particularly diarrhea and stomatitis (42% *vs*. 20%), as well as of anti-EGFR-related AEs (76% *vs*. 42%), was found with 5FU/LV+panitumumab compared to that with panitumumab alone (9).

Drawing from this puzzling evidence, the present study retrospectively assessed the effectiveness and safety outcomes of the different post-induction strategies adopted in clinical practice in a selected population of patients with left-sided *RAS* and *BRAF* wild-type mCRC.

The first result to be discussed is the relatively low rate of patients undergoing a chemotherapy-only maintenance treatment, particularly with 5FU/LV alone, as compared to patients treated with 5FU/LV+anti-EGFR and anti-EGFR alone. This is in line with the scarce evidence previously discussed, as, to date, no evidence supports the use of 5FU/LV alone as maintenance after an anti-EGFR-based induction regimen. In this respect, in the randomized phase 2 PanaMa trial comparing 5FU/LV+panitumumab *vs*. 5FU/LV alone as maintenance strategies in *RAS* wild-type mCRC, the PFS of maintenance therapy was significantly improved with 5FU/LV+panitumumab (8.8 *vs*. 5.7 months), with a trend toward better OS (28.7 *vs*. 25.7 months) (10).

With the exception of a higher tumor burden and a more extensive use of FOLFIRI and cetuximab in the non-maintenance cohort compared to the maintenance cohorts, no significant differences were found regarding the baseline characteristics and prognostic factors, which were fairly balanced among the numerically larger cohorts (i.e., 5FU/LV+anti-EGFR, anti-EGFR, and non-maintenance). The higher tumor burden might have negatively affected the clinical histories and steered the clinicians’ choice toward not de-escalating the treatment. The median number of induction cycles was also balanced between the cohorts. These results are consistent with previously summarized literature data, as the maintenance (10–12) or intermittent (13, 14) strategies have been mainly investigated in patients treated with an oxaliplatin-based chemotherapy backbone following a 6- to 12-cycle (i.e., about 3–6 months) induction in order to reduce the incidence of peripheral neuropathy. The higher proportion of patients treated with a FOLFIRI chemotherapy backbone up to disease progression, unacceptable toxicity, or patient decision in the non-maintenance compared to the maintenance cohorts, together with the higher incidence of non-hematological and hematological AEs, including diarrhea and neutropenia, emphasized the issue of dealing with irinotecan-related cumulative toxicities and the need for comparative trials of post-induction management in this setting. In this respect, the results of the ongoing phase 2 IMPROVE trial (NCT04425239), aimed at comparing intermittent first-line FOLFIRI-panitumumab *vs*. the same regimen given continuously, and those of the ongoing randomized phase 3 ERMES trial (22), aimed at comparing FOLFIRI–cetuximab *vs*. maintenance cetuximab following FOLFIRI–cetuximab, both in a population of patients with unresectable *RAS/BRAF* wild-type mCRC, are awaited.

In our study, a clinically relevant and statistically significant benefit was observed for patients treated with 5FU/LV+anti-EGFR maintenance over the non-maintenance strategy, while maintenance treatment with anti-EGFR alone achieved less clear results. This differential advantage is confirmed by the progressive reduction in the relative risk of disease progression or death. This survival benefit was associated with a higher incidence of hematological and non-hematological AEs, other than paronychia/nail disorders, which usually occur after several weeks of treatment with anti-EGFR, particularly in association with fluoropyrimidines (23). These results are consistent with the existing literature, corroborating the hypothesis that patients gain more in terms of PFS and OS from a maintenance approach than from a “stop-and-go” strategy (13), with an even greater benefit for FU/LV+anti-EGFR compared to anti-EGFR alone, particularly in patients affected by left-sided *RAS*/*BRAF* wild-type mCRC, at the price of a slightly higher incidence of manageable toxic effects (9, 10).

As expected, the majority of patients with disease progression underwent an antiangiogenic-based second-line treatment, according to recommendations from national and international guidelines (24, 25) and retrospective experiences (26). The small number of patients who were reintroduced to an anti-EGFR-based regimen at disease progression, particularly in the non-maintenance cohort, limited further assessment of the value of a “stop-and-go” strategy, which might be effective in patients with rapid and deep tumor responses, with low tumor burden, especially, but not only, if converted to radical surgery following an anti-EGFR-containing first-line induction, or in those patients who experienced severe or disabling skin toxicity (27). In the PaNama trial, reinduction therapy was more active and effective in patients who had received FU/LV compared to those who received FU/LV+anti-EGFR (ORR = 34.7% *vs*. 8.9%, PFS = 6.3 *vs*. 3.8 months, respectively). On the other hand, preclinical and clinical evidence suggests a potential role of anti-EGFR reintroduction beyond the second-line, particularly in patients selected with liquid biopsy (28–31).

The retrospective nature of the study, with its inherent selection bias and not-on-purpose data collection, and the small sample size of the cohorts are some of the limitations that might have affected the results of this study. As mentioned, confounding by indication could have played a role in the observed inter-cohort differences. Although we may not draw definitive conclusions from our study, its interesting findings can be considered as preliminary and hypothesis-generating. Moreover, the study provided a snapshot of the real-life attitude of clinicians toward the post-induction strategy for patients with unresectable left-sided *RAS*/*BRAF* wild-type mCRC treated with anti-EGFR-based doublet first-line induction and contextualized it within the relative scantiness of literature data. In this respect, prospective observational studies, with more homogeneous inclusion criteria and patient characteristics, addressing the reasons leading to maintenance strategies in a real-life setting are certainly desirable.

## Conclusion

The ideal maintenance strategy should preserve the obtained response over time by administering an appropriate less toxic regimen, preserving the quality of life of patients without compromising treatment efficacy. In our real-life cohort, maintenance with 5FU/LV+anti-EGFR seems to be the most widely adopted, as well as a safe and effective regimen in patients with unresectable left-sided *RAS*/*BRAF* wild-type mCRC treated with an anti-EGFR-based doublet first-line induction regimen. However, intermittent or continuous treatment strategies might still be options in a case-by-case evaluation based on both patient and disease characteristics and response and safety to induction treatment.

## Data Availability Statement

The raw data supporting the conclusions of this article will be made available by the authors, without undue reservation.

## Ethics Statement

The studies involving human participants were reviewed and approved by Comitato Etico delle Province di L’Aquila e Teramo. The patients/participants provided written informed consent to participate in this study.

## Author Contributions

All authors contributed to the publication according to the ICMJE guidelines for authorship. All authors read and approved the manuscript and agree to be accountable for all aspects of the research in ensuring that the accuracy or integrity of any part of the work are appropriately investigated and resolved. All authors contributed to the article and approved the submitted version.

## Conflict of Interest

AlP reported receiving advisory board fees from GSK. AC reported receiving consulting/advisory board fees from AstraZeneca, MSD, Roche, and BMS and speakers’ fee from Novartis, Astellas, MSD, and AstraZeneca. AnP reported receiving fees from Eli-Lilly, MSD, and Servier. RG reported receiving fees from Amgen and Servier and advisory board fees from Amgen, Servier, Bayer, and Merck-Serono.

The remaining authors declare that the research was conducted in the absence of any commercial or financial relationships that could be construed as a potential conflict of interest.

## Publisher’s Note

All claims expressed in this article are solely those of the authors and do not necessarily represent those of their affiliated organizations, or those of the publisher, the editors and the reviewers. Any product that may be evaluated in this article, or claim that may be made by its manufacturer, is not guaranteed or endorsed by the publisher.

## References

[1] Tournigand C Cervantes A Figer A Lledo G Flesch M Buyse M. OPTIMOX1: A Randomized Study of FOLFOX4 or FOLFOX7 With Oxaliplatin in a Stop-and-Go Fashion in Advanced Colorectal Cancer—A GERCOR Study. J Clin Oncol (2006) 24(3):394–400. doi: 10.1200/JCO.2005.03.0106 16421419

[2] Chibaudel B Maindrault-Goebel F Lledo G Mineur L André T Bennamoun M. Can Chemotherapy be Discontinued in Unresectable Metastatic Colorectal Cancer? The GERCOR OPTIMOX2 Study. J Clin Oncol (2009) 27(34):5727–33. doi: 10.1200/JCO.2009.23.4344 19786657

[3] Hegewisch-Becker S Graeven U Lerchenm̈ller CA Killing B Depenbusch R Steffens CC. Maintenance Strategies After First-Line Oxaliplatin Plus Fluoropyrimidine Plus Bevacizumab for Patients With Metastatic Colorectal Cancer (AIO 0207): A Randomised, Non-Inferiority, Open-Label, Phase 3 Trial. Lancet Oncol (2015) 16(13):1355–69. doi: 10.1016/S1470-2045(15)00042-X 26361971

[4] Koeberle D Betticher DC von Moos R Dietrich D Brauchli P Baertschi D. Bevacizumab Continuation *Versus* No Continuation After First-Line Chemotherapy Plus Bevacizumab in Patients With Metastatic Colorectal Cancer: A Randomized Phase III Non-Inferiority Trial (SAKK 41/06). Ann Oncol (2015) 26(4):709–14. doi: 10.1093/annonc/mdv011 25605741

[5] Simkens LH van Tinteren H May A ten Tije AJ Creemers GJ Loosveld OJ. Maintenance Treatment With Capecitabine and Bevacizumab in Metastatic Colorectal Cancer (CAIRO3): A Phase 3 Randomised Controlled Trial of the Dutch Colorectal Cancer Group. Lancet (2015) 385(9980):1843–52. doi: 10.1016/S0140-6736(14)62004-3 25862517

[6] Van Cutsem E Cervantes A Adam R Sobrero A Van Krieken JH Aderka D. ESMO Consensus Guidelines for the Management of Patients With Metastatic Colorectal Cancer. Ann Oncol (2016) 27(8):1386–422. doi: 10.1093/annonc/mdw235 27380959

[7] Arnold D Lueza B Douillard JY Peeters M Lenz HJ Venook A. Prognostic and Predictive Value of Primary Tumour Side in Patients With RAS Wild-Type Metastatic Colorectal Cancer Treated With Chemotherapy and EGFR Directed Antibodies in Six Randomized Trials. Ann Oncol (2017) 28(8):1713–29. doi: 10.1093/annonc/mdx175 PMC6246616 28407110

[8] Parisi A Porzio G Cannita K Venditti O Avallone A Filippi R. Clinicians' Attitude to Doublet Plus Anti-EGFR *Versus* Triplet Plus Bevacizumab as First-Line Treatment in Left-Sided RAS and BRAF Wild-Type Metastatic Colorectal Cancer Patients: A Multicenter, "Real-Life", Case-Control Study. Clin Colorectal Cancer (2021) S1533-0028(21):00068–2. doi: 10.1016/j.clcc.2021.07.003. S1533-0028(21)00068-2. 34380594

[9] Pietrantonio F Morano F Corallo S Miceli R Lonardi S Raimondi A. Maintenance Therapy With Panitumumab Alone *vs* Panitumumab Plus Fluorouracil-Leucovorin in Patients With RAS Wild-Type Metastatic Colorectal Cancer: A Phase 2 Randomized Clinical Trial. JAMA Oncol (2019) 5(9):1268–75. doi: 10.1001/jamaoncol.2019.1467 PMC6613306 31268481

[10] Modest DP Karthaus M Fruehauf S Graeven U Müller L König AO. Panitumumab Plus Fluorouracil and Folinic Acid *Versus* Fluorouracil and Folinic Acid Alone as Maintenance Therapy in RAS Wild-Type Metastatic Colorectal Cancer: The Randomized PANAMA Trial (AIO KRK 0212). J Clin Oncol (2021), JCO2101332. doi: 10.1200/JCO.21.01332 34533973 PMC8683209

[11] Aranda E García-Alfonso P Benavides M Sánchez Ruiz A Guillén-Ponce C Safont MJ. Spanish Cooperative Group for the Treatment of Digestive Tumours (TTD). First-Line mFOLFOX Plus Cetuximab Followed by mFOLFOX Plus Cetuximab or Single-Agent Cetuximab as Maintenance Therapy in Patients With Metastatic Colorectal Cancer: Phase II Randomised MACRO2 TTD Study. Eur J Cancer (2018) 101:263–72. doi: 10.1016/j.ejca.2018.06.024 30054049

[12] Munemoto Y Nakamura M Takahashi M Kotaka M Kuroda H Kato T. SAPPHIRE: A Randomised Phase II Study of Planned Discontinuation or Continuous Treatment of Oxaliplatin After Six Cycles of Modified FOLFOX6 Plus Panitumumab in Patients With Colorectal Cancer. Eur J Cancer (2019) 119:158–67. doi: 10.1016/j.ejca.2019.07.006 31445198

[13] Wasan H Meade AM Adams R Wilson R Pugh C Fisher D. COIN-B Investigators. Intermittent Chemotherapy Plus Either Intermittent or Continuous Cetuximab for First-Line Treatment of Patients With KRAS Wild-Type Advanced Colorectal Cancer (COIN-B): A Randomized Phase 2 Trial. Lancet Oncol (2014) 15(6):631–9. doi: 10.1016/S1470-2045(14)70106-8 PMC4012566 24703531

[14] Pfeiffer P Sorbye H Qvortrup C Karlberg M Kersten C Vistisen K. Maintenance Therapy With Cetuximab Every Second Week in the First-Line Treatment of Metastatic Colorectal Cancer: The NORDIC-7.5 Study by the Nordic Colorectal Cancer Biomodulation Group. Clin Colorectal Cancer (2015) 14(3):170–6. doi: 10.1016/j.clcc.2015.03.002 25956187

[15] Eisenhauer EA Therasse P Bogaerts J Schwartz LH Sargent D Ford R. New Response Evaluation Criteria in Solid Tumours: Revised RECIST Guideline (Version 1.1). Eur J Cancer (2009) 45:228–47. doi: 10.1016/j.ejca.2008.10.026 19097774

[16] Woolley KK. How Variables Uncorrelated With the Dependent Variable Can Actually Make Excellent Predictors: The Important Suppressor Variable Case. Southwest Educational Research Association Annual Meeting Proceedings (1997). Available at: https://eric.ed.gov/?id=ED407420.

[17] Thompson FT Levine DU. Examples of Easily Explainable Suppressor Variables in Multiple Regression Research. Multiple Linear Regression Viewpoints (1997) 24:11–3.

[18] “Stopping Stepwise: Why Stepwise Selection is Bad and What You Should Use Instead”. Available at: https://towardsdatascience.com/stopping-stepwise-why-stepwise-selection-is-bad-and-what-you-should-use-instead90818b3f52df.

[19] Köhne CH Grothey A Bokemeyer C Bontke M Aapro M. Chemotherapy in Elderly Patients With Colorectal Cancer. Ann Oncol (2001) 12(4):435–42. doi: 10.1023/A:1011170808734 11398873

[20] Yang Y Wang G He J Ren J Wu J Zhang J. Gender Differences in Colorectal Cancer Survival: A Meta-Analysis. Int J Cancer (2017) 141:1942–9. doi: 10.1002/ijc.30827 28599355

[21] Köhne CH Cunningham D Di Costanzo F Glimelius B Blijham G Aranda E. Clinical Determinants of Survival in Patients With 5-Fluorouracil-Based Treatment for Metastatic Colorectal Cancer: Results of a Multivariate Analysis of 3825 Patients. Ann Oncol (2002) 13(2):308–17. doi: 10.1093/annonc/mdf034 11886010

[22] Pinto C Normanno N Orlandi A Fenizia F Damato A Maiello E. Phase III Study With FOLFIRI + Cetuximab *Versus* FOLFIRI + Cetuximab Followed by Cetuximab Alone in RAS and BRAF WT mCRC. Future Oncol (2018) 14(14):1339–46. doi: 10.2217/fon-2017-0592 29846100

[23] Lacouture ME Sibaud V Gerber PA van den Hurk C Fernández-Peñas P Santini D. Prevention and Management of Dermatological Toxicities Related to Anticancer Agents: ESMO Clinical Practice Guidelines. Ann Oncol (2021) 32(2):157–70. doi: 10.1016/j.annonc.2020.11.005 33248228

[24] Linee Guida AIOM TUMORI DEL COLON. Available at: https://www.aiom.it/wp-content/uploads/2020/10/2020_LG_AIOM_Colon.pdf.

[25] Van Cutsem E Cervantes A Adam R Sobrero A Van Krieken JH Aderka D. ESMO Consensus Guidelines for the Management of Patients With Metastatic Colorectal Cancer. Ann Oncol (2016) 27(8):1386–422. doi: 10.1093/annonc/mdw235 27380959

[26] Parisi A Cortellini A Cannita K Venditti O Camarda F Calegari MA. Evaluation of Second-Line Anti-VEGF After First-Line Anti-EGFR Based Therapy in RAS Wild-Type Metastatic Colorectal Cancer: The Multicenter "SLAVE" Study. Cancers (Basel) (2020) 12(5):1259. doi: 10.3390/cancers12051259 PMC7281759 32429380

[27] Petrelli F Pietrantonio F Cremolini C Di Bartolomeo M Coinu A Lonati V. Early Tumour Shrinkage as a Prognostic Factor and Surrogate End-Point in Colorectal Cancer: A Systematic Review and Pooled-Analysis. Eur J Cancer (2015) 51(7):800–7. doi: 10.1016/j.ejca.2015.02.011 25794604

[28] Ciardiello F Normanno N Martinelli E Troiani T Pisconti S Cardone C. Cetuximab Continuation After First Progression in Metastatic Colorectal Cancer (CAPRI-GOIM): A Randomized Phase II Trial of FOLFOX Plus Cetuximab *Versus* FOLFOX. Ann Oncol (2016) 27(6):1055–61. doi: 10.1093/annonc/mdw136 27002107

[29] Parseghian C Loree J Morris V Liu X Clifton K Napolitano S. Anti-EGFR-Resistant Clones Decay Exponentially After Progression: Implications for Anti-EGFR Re-Challenge. Ann Oncol (2019) 30:243–9. doi: 10.1093/annonc/mdy509 PMC6657008 30462160

[30] Parisi A Porzio G Pulcini F Cannita K Ficorella C Mattei V. What Is Known About Theragnostic Strategies in Colorectal Cancer. Biomedicines (2021) 9(2):140. doi: 10.3390/biomedicines9020140 33535557 PMC7912746

[31] Ciardiello D Martini G Famiglietti V Napolitano S De Falco V Troiani T. Biomarker-Guided Anti-Egfr Rechallenge Therapy in Metastatic Colorectal Cancer. Cancers (Basel) (2021) 13(8):1941. doi: 10.3390/cancers13081941 33920531 PMC8073594

