# Supplementary material for: Post-Induction Management in Patients With Left-Sided RAS and BRAF Wild-Type Metastatic Colorectal Cancer Treated With First-Line Anti-EGFR-Based Doublet Regimens: A Multicentre Study
Source: Front Oncol. 2021 Oct 27;11:712053. doi: 10.3389/fonc.2021.712053 (PMC8579084; doi:10.3389/fonc.2021.712053)
Supplement: Supplementary file 1 [file DataSheet_1.docx]

| **Institution** | **Department** |
| --- | --- |
| St. Salvatore Hospital, University of L’Aquila, L’Aquila | Medical Oncology |
| Policlinico Universitario Agostino Gemelli, IRCCS - Comprehensive Cancer Center, Rome, Italy | Medical Oncology |
| ASST Sette Laghi, Ospedale di Circolo e Fondazione Macchi, Varese, Italy | Medical Oncology |
| Campus Bio-Medico University, Rome, Italy | Medical Oncology |
| Department of Oncology and Hematology, Division of Oncology, University Hospital of Modena, Modena, Italy | Division of Oncology |
| P.O. “Vito Fazzi”, Lecce, Italy | Medical Oncology |
| Clinical Oncology Unit, S.S. Annunziata Hospital, Chieti, Italy | Clinical Oncology Unit |
| Clinica Oncologica e Centro Regionale di Genetica Oncologica, Università Politecnica delle Marche, AOU Ospedali Riuniti-Ancona, Italy | Medical Oncology |
| Medical Oncology Unit, Ospedale del Mare, Naples, Italy | Medical Oncology Unit |
| Città della Salute e della Scienza di Torino, Turin, Italy | Medical Oncology 1 |
| Candiolo Cancer Institute - FPO-IRCCS, Candiolo (TO), Italy | Medical Oncology |
| Medical Oncology, ASL TO4, Ospedale Civile di Ivrea, Ivrea, Turin, Italy | Medical Oncology |
| Department of Medical Oncology, INCLIVA Biomedical Research Institute, University of Valencia, 46010 Valencia, Spain | Medical Oncology |
| Experimental Clinical Abdominal Oncology Unit, Istituto Nazionale Tumori-IRCCS-Fondazione G.Pascale, Naples, Italy | Medical Oncology |
| Department of Precision Medicine, Università della Campania "Luigi Vanvitelli", 80131 Naples, Italy | Medical Oncology |
| IRCCS Istituto Dermopatico dell'Immacolata (IDI), Rome, Italy | Medical Oncology |
| Tor Vergata University Hospital, Viale Oxford, 81, 00133, Rome, Italy | Medical Oncology |
| Oncology Unit, Sant’Andrea Hospital, Rome, Italy | Medical Oncology |
| Fondazione IRCCS Ca' Granda Ospedale Maggiore Policlinico, Milan, Italy | Medical Oncology |
| Medical Oncology Unit, University Hospital of Parma, Via Gramsci 14, 43126 Parma, Italy | Medical Oncology |
| Santa Maria Goretti Hospital, Latina, Italy | Medical Oncology |
| UOC Territorial Oncology - AUSL Latina-CdS Aprilia – University of Rome “Sapienza”, Taly | Medical Oncology |

**Supplementary file.** List of participating centres.
